# Seepage stability analysis of geogrid reinforced tailings dam

**DOI:** 10.1038/s41598-024-52472-y

**Published:** 2024-01-20

**Authors:** Changbo Du, Han Tao, Fu Yi

**Affiliations:** 1 College of Civil Engineering, Liaoning Technical University, Fuxin, 123000 China; 2 Beijing Jingneng Geological Engineering Co., Ltd, Beijing, 102300 China

**Keywords:** Civil engineering, Solid Earth sciences

## Abstract

To investigate the influence of a geogrid-reinforced tailings dam on the seepage stability of the dam body, this paper was based on the field test of a reinforced tailings accumulation dam. The study utilized the finite element strength reduction method to simulate the stability of the main dam of the Fengshuigou tailings reservoir under different seepage conditions using ABAQUS software. Additionally, the paper discussed the impact of conventional heightening, dry beach length, and geogrid reinforcement on the position and safety factor of the saturation line of the dam body. The results showed that when the dam body was raised, the saturation line rose by 2.8–5.3 m, resulting in a decrease in the safety factor. The geogrid effectively reduced the height of the saturation line in the tailings dam. In comparison to the unreinforced condition (dam heightening), the saturation line of the tailings dam decreased by 0.9–2.8 m under the local reinforcement condition and by 3.2–12.5 m under the overall reinforcement condition. The geogrid significantly improved the stability of the tailings dam. Furthermore, under the local reinforcement condition, the safety factor of the dam increased by 3.8–5.5%, and under the overall reinforcement condition, it increased by 35.9–42.9%, when compared to the unreinforced condition. Increasing the dry beach length improved the stability of the tailings dam, and under normal working conditions, the safety of the tailings dam was much higher than under the minimum dry beach condition. These results served as a reference for the design of the dam and the new tailings reservoir, laying a foundation for the sustainable development and environmental protection of the tailings pond.

## Introduction

The stability of the tailings dam was closely related to the safe operation of the whole tailings reservoir. According to a review of relevant information^1^, the failure of a tailings dam seriously threatened the safety, life, and property of downstream inhabitants, as well as polluted and damaged the ecological environment. For example, in 2019, the Córrego do Feijão tailings dam I in Brumadinho, Minas Gerais, broke and released a large amount of tailings, resulting in the deaths of 660 people and polluting downstream rivers^2^. In 1985, a dam failure in a tailings pond in Stava, northern Italy, resulted in 268 deaths and significant economic losses^3^. With governments’ increasing attention to the safety of tailings ponds, the overall safety level has been significantly improved. However, the problem of heavy rainfall climate has gradually become the main factor causing the tailings dam to break^4^. Because the permeability of the tailings dam was greatly affected by the seepage field, the location of the saturation line in the seepage field was closely related to the length of the dry beach and the rainfall intensity^5^.

To make an accurate evaluation of the stability of the tailings dam, similar physical model tests and numerical simulations were usually used to evaluate. For the physical model, selecting reasonably similar materials was particularly important for the test results. However, choosing similar materials often involves many subject knowledge and was more complicated. In contrast, numerical simulation has gradually become an important method to evaluate the stability of tailings dams. At present, many scholars have used the finite element method to establish two-dimensional or three-dimensional models to carry out research^6,7^. Lu et al.^8^ proposed that proper simplification and generalization of complex terrain in three-dimensional (3D) numerical calculation had little impact on the results and could meet the accuracy requirements. Based on the stochastic limit equilibrium method, Mafi et al.^9^ analyzed the dam’s stability with three different slopes. Dastpak et al.^10^ analyzed the stability of geosynthetic reinforced slopes based on non-circular certainty and randomness. Aroni Hesari et al.^11^ used the horizontal slice method to study the seismic internal stability of geosynthetic reinforced soil slopes. Fatehi et al.^12^ used the pseudo-static method to examine the stability of reinforced slopes under seismic load. Doğan and Güllü^13^ proposed a 3D voxel model generation method for finite element structural analysis. Wang et al.^14^ analyzed the stability of tailings dams under dry–wet cycles and proposed an effective calculation method for the saturation line of tailings dams under dry–wet cycles. Wang^15^ analyzed the seepage condition of the dam body under the current elevation and the final design elevation of the tailings pond through theoretical analysis and numerical calculation. Zhang et al.^16^ analyzed the influence of different dry beach and upstream-side slope ratios on the seepage stability of the tailings dam. Naeini et al.^17^ used SIGMA/W software to analyze the stress-pore pressure coupling. It can be seen from the above research results that the limit equilibrium method was mainly used to solve the safety factor of the tailings dam. When the limit equilibrium method was used to analyze the influence of pore water pressure on the stability of the tailings dam, the pore water pressure was treated as zero in the case of an unsaturated area, ignoring its influence. At the same time, many factors affected the stability of the tailings dam, but most were related to saturation line, dry beach length, and pore pressure. The research on seepage stability of tailings dams reinforced by geogrid was relatively weak.

Given how to improve the tailings dam stability, many scholars conducted relevant research, mainly including the following aspects: First, they used geosynthetics or fibers to reinforce tailings dams. It included geogrid, geotextile, geomembrane, geobag, various fibers, etc. Xiao^18^ used a Geo-slope to analyze the geosynthetic-reinforced Jinshandian tailings dam. The results showed that reinforcement could effectively improve the stability of the fill dam. Zhou et al.^19^ studied tailing-geotextile composites, analyzed their permeability and physical properties, and generalized the composite model. Li et al.^20^ established a stability analysis method based on limit equilibrium theory to simulate the stability of tailings dams using geotextile bags. Zheng et al.^21^ used basalt fiber to reinforce a tailings dam and improved its stability. Second, they used chemical reagents or biological consolidation effectively to improve the stability of the dam. Yang et al.^22^ used polyacrylamide to enhance the dynamic characteristics of tailings, thereby improving the stability of tailings dams. Lu et al.^23^ used microbially induced calcite precipitation to reinforce tailings. Sun et al.^24^ studied the mechanical reinforcement effect of a tailings dam slope under four conditions: goosegrass, vetiver grass, clover, and bare slope. The results showed that vetiver grass was more conducive to dam reinforcement. The third aspect was to improve the dam’s stability from the perspective of anti-seepage, mainly involving material properties and foundation treatment. In addition to using anti-seepage membranes, the ground could be improved by grouting to have low permeability, low settlement, and high bearing capacity^25,26^. At the same time, when the tailings dam produced a seepage channel, it could be blocked by grouting^27,28^. The fourth approach was to use online monitoring technology to monitor the dam in real-time. Olivier et al.^29^ used environmental-seismic noise to monitor the stability of tailings dams, which provided a valuable tool for the remote monitoring of the structural stability of tailings dams. In summary, many scholars have conducted a lot of research on the seepage stability and dam reinforcement of tailings dams. Although some achievements have been made, the study mainly focused on the influence of a single factor. The research on the impact of geogrid-reinforced tailings dams under multi-factor coupling still needs further discussion.

**Figure 1:**
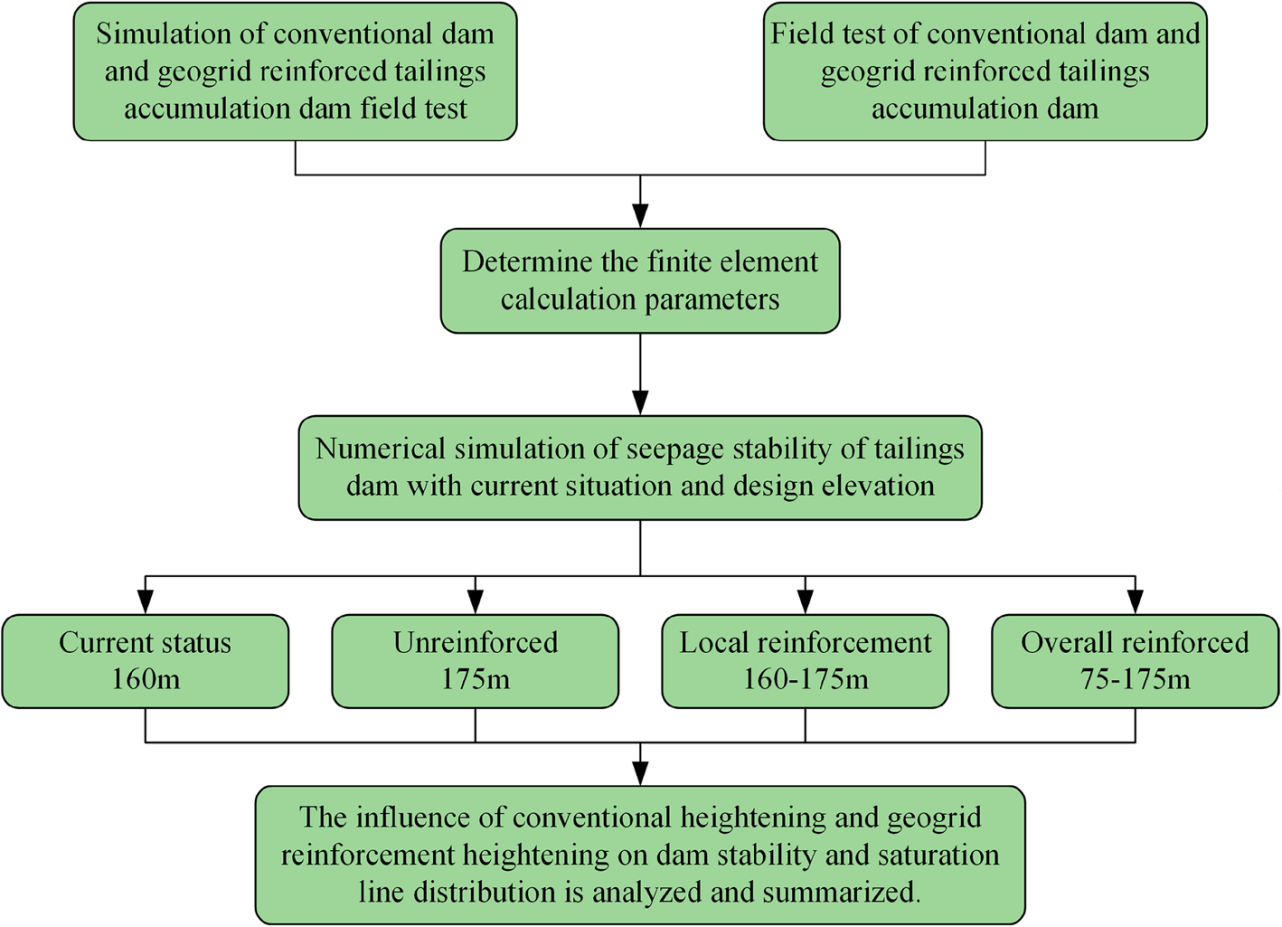
Flowchart of the research process utilized in this study.

In this study, based on the previously conducted field test^30^ of the Fengshuigou tailings reservoir, reasonable model parameters were selected by ABAQUS finite element software, and the conventional accumulation dam and geogrid reinforced accumulation dam of the field test were simulated and calculated, respectively. By comparing the test and simulation results, the accuracy of the model parameter inversion was assessed. Based on the fluid–solid coupling theory and the finite element strength reduction method, two tailings dam models with two tailings dam elevations (current and design elevations) and two reinforcement types (geogrid local and overall reinforcement) were established. The stability of the tailings dam under three dry beach lengths (minimum dry beach, flood operation, and normal operation) was calculated. The influence of dry beach length, conventional heightening, and geogrid reinforcement on the saturation line and safety factor of the dam body was studied (see Fig. 1 for the specific process), and reference suggestions were made for the engineering application of reinforced tailings reservoirs.

## Field test simulation and determination of permeability coefficient

This study was based on field tests of reinforced tailings stacking dams in the Fengshuigou tailings pond^30^. ABAQUS simulations were performed for the field tests, a finite element model was established, an inverse trial analysis was performed, and the simulation results were compared with the field monitoring results to obtain a more reasonable calculation model and calculation parameters. On this basis, we analyzed the influence law of geogrid reinforcement on the pore pressure (saturation line), internal pressure, and deformation of stacked dams.

### Field test overview

**Figure 2:**
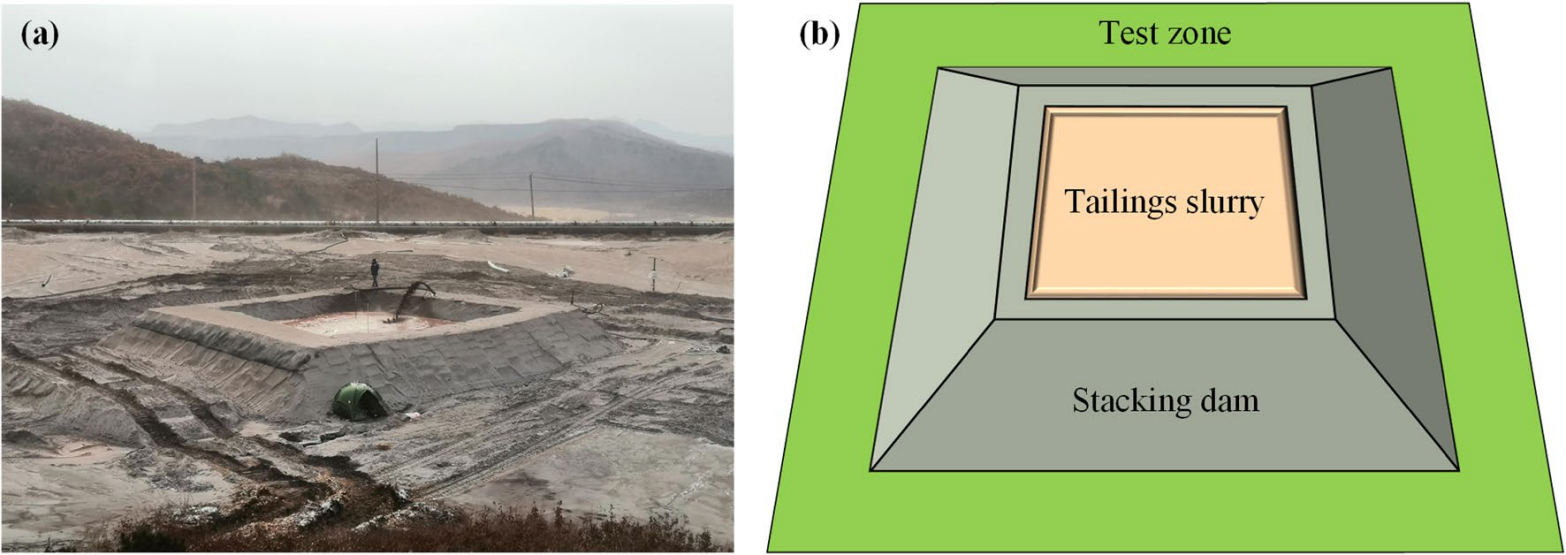
Field test of reinforced tailings dam: (**a**) Test site diagram; (**b**) Three-dimensional diagram.

The field test of the reinforced accumulation dam was conducted on the dry beach surface of the No.5 auxiliary dam of the Fengshuigou tailings reservoir, and the side length of the specific test area was approximately 15 m. After excavating about 1 m shallow pits in the test area, a 4 m high accumulation dam was built, including conventional dams, and two-way geogrid reinforced dam. The outer slope ratio of the surrounding accumulation dam was 1:1, and the width of the dam crest was 3 m. The field test of the reinforced tailings dam is shown in Fig. 2a, and a 3D schematic diagram is shown in Fig. 2b. The performance parameters of the geogrid used are listed in Table 1.

**Table 1:** Geogrid performance parameters.

EGA30 Geogrid mechanical parameters	Performance index
Mesh size (length × width)/mm	12.7 × 12.7
Fracture strength/(kN/m)	
Diametral	30
Latitudinal	30
Elongation at break ≥ /%	
Diametral	4
Latitudinal	4

### Establishment of finite element model

#### Model calculation parameters

In this study, both the stacked and geogrid dams were modeled as solids in the finite element calculations. The Mohr–Coulomb plastic model was utilized for the stacked dam and subgrade, and the elastic model was applied for the geogrid. The embedded constraint simulated the interaction between the tailings sand and the geogrid. Considering the excessive contact surface of the model, the calculation quickly became non-convergent and held calculation time; thus, it was assumed that no relative sliding occurred between the tailing sand and the geogrid. The strength reduction method was used to calculate the stability of the dam. A different strength reduction coefficient $F_{r}$ was assumed in the calculation, and the finite element analysis was carried out according to the strength parameters after reduction to observe whether the calculation is convergent. In the calculation process, $F_{r}$ (using dichotomy) was continuously increased, and the strength reduction factor $F_{r}$ at the critical failure was the slope stability safety factor $F_{s}$. The shear strength parameters after reduction are shown in Eqs. (1) and (2):

1 $$c_{m} = c/F_{r}$$

2 $$\varphi_{m} = \arctan ((\tan \varphi )/F_{r})$$

where $c$ and $\varphi$ were the shear strength of the soil; $c_{m}$ and $\varphi_{m}$ were the shear strength required for the actual; $F_{r}$ was the strength reduction factor.

**Figure 3:**
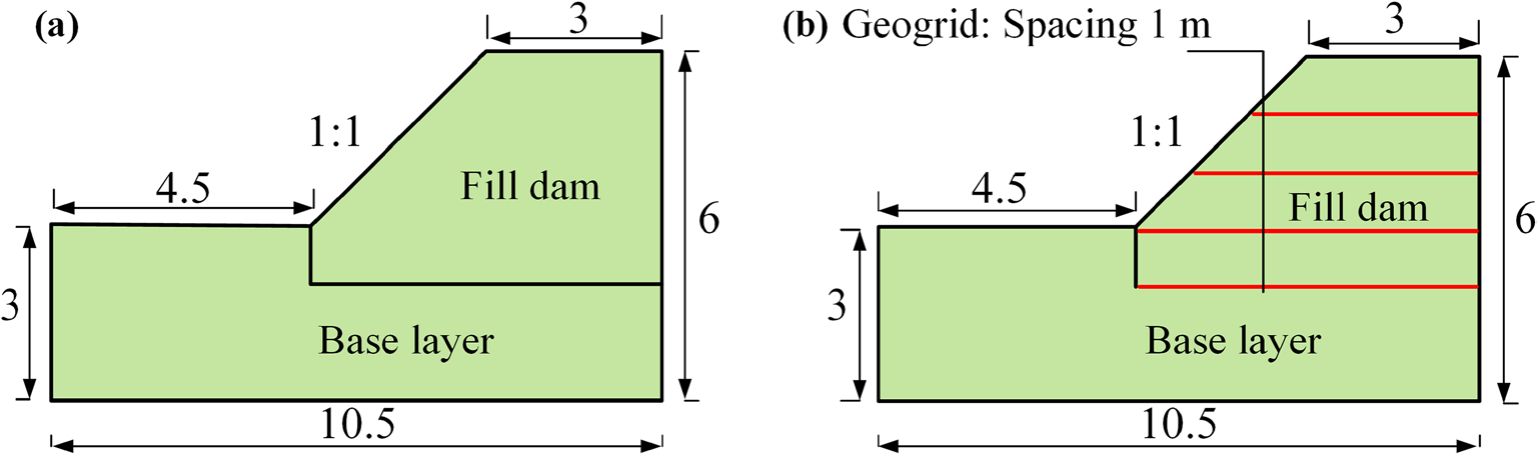
Cross section of tailings accumulation dam (unit: m): (**a**) Conventional dam; (**b**) Geogrid reinforced dam.

Only gravity loads were considered in the model calculation, without considering external factors such as overload and temperature. The cross-sectional view of the model of the unreinforced and gridded reinforced pile dam is shown in Fig. 3. The laying of the geogrid is divided into four layers: the first layer is laid on the bottom of the accumulation dam; the second, third, and fourth layers are laid every 1 m in the middle; and the top surface is not laid with reinforcement. Based on the engineering investigation report and with reference to the literature^31^, the model calculation parameters are listed in Table 2.

**Table 2:** Model calculation parameters.

Material	Unit weight/(kN·m^−3^)	Elastic modulus/kPa	Cohesion/kPa	Internal friction angle/°	Poisson’s ratio
Accumulation dam	17.4	1.6 × 10^5^	1.5	33.4	0.25
Subbase	20.0	4.5 × 10^6^	10	34.6	0.30
Geogrid	0.78	2.5 × 10^9^	–	–	0.29

#### Model boundary conditions

Simulations were performed to reduce the influence of boundary effects by expanding the boundaries in the left and lower directions of the tailings dam model. Based on data from the literature^32^, the distance from the left boundary to the toe of the slope of the computational model was taken as 1.5 times the slope height, and the bottom of the slope extended downward by 1 times the slope height. The boundary conditions were set as follows: the bottom boundary of the stacked dam was constrained in the x, y, and z directions, the upper and lower boundaries were constrained in the x direction only, the boundaries of both sides of the section were constrained in the y direction only, and the top of the slope and the slope surface were not constrained. The pore pressure boundary on the right side of the model was determined by the relationship between the water level in the reservoir of the surrounding pile dam and time, and the slope and foot of the slope were set as the free seepage section boundary.

#### Mesh generation of model

**Figure 4:**
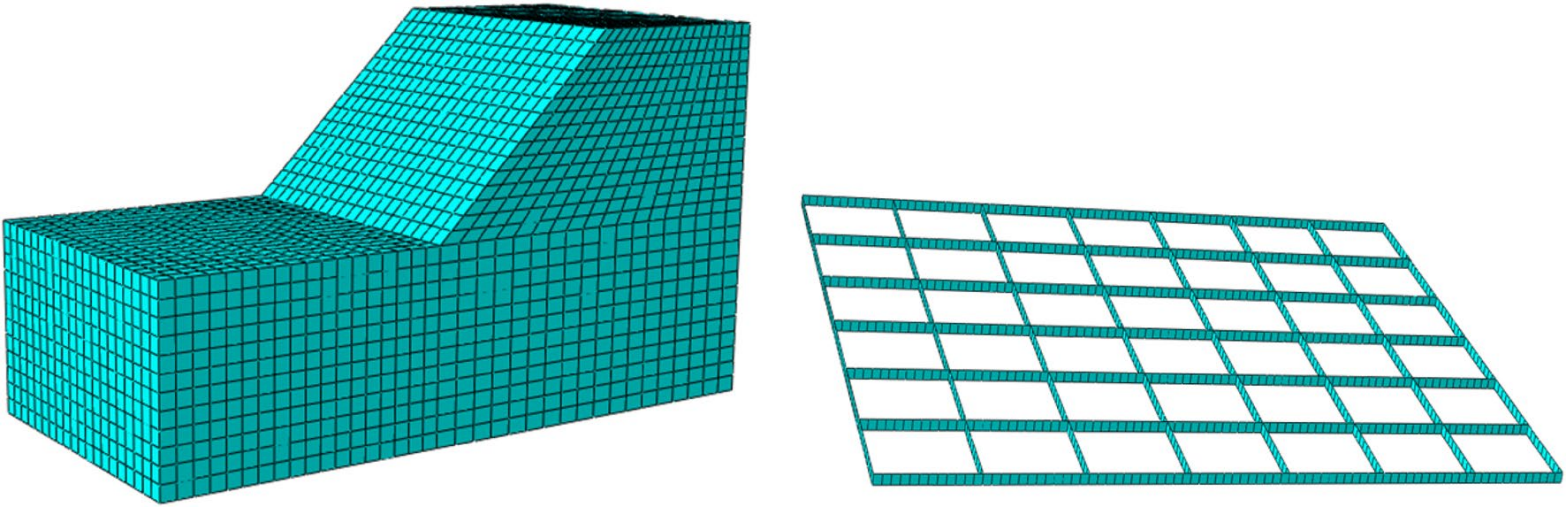
Unit mesh model of tailings accumulation dam.

CPE8P cells were used to mesh the two types of pile dams, and 1298 soil cells were used after dividing the cells of the conventional pile dam. Then, the four layers of geogrid reinforcement were divided into 438, 589, 759, and 759 cells. The stacked dam cells and geogrid cells are shown in Fig. 4. In the finite element simulation, we also simulated the discharge slurry time of 3 h, so the numerical simulation results showed a sharp decline that recovered within 3–5 h.

### Penetration coefficient inversion trial calculation

#### Range of permeability coefficient

A permeability coefficient test was conducted out to obtain a permeability coefficient close to the actual operating conditions of the project. The tailing material parameter affecting the measured water level was the permeability coefficient of each soil layer; therefore, the permeability coefficient was selected for stochastic inversion. The parameters involved in this permeability coefficient inversion included the permeability coefficients of the stacked dam body and geogrid. According to the report provided by the geogrid manufacturer and the related literature^30^, the range of the permeability coefficient and the initial value of the trial calculation were determined, as shown in Table 3.

**Table 3:** Value range of permeability coefficient.

Partition	Value range/(m s^−1^)		Trial initial value
	Maximum	Minimum	
Accumulation dam body	2.4 × 10^−4^	2.4 × 10^−6^	2.0 × 10^−5^
Geogrid	1.1 × 10^−3^	1.1 × 10^−5^	1.0 × 10^−4^

In this section, the positive algorithm was used as the basic method for inversion parameter trial calculation, combined with ABAQUS software for field test simulation. According to the two types of monitoring data, pore and soil pressure, the actual monitoring values of pore and soil pressure were analyzed and compared with the simulated calculated values, and the distribution law was derived. The model parameters were adjusted and calculated individually until the difference between them met the accuracy requirements, thus determining the infiltration parameters that could be used.

**Figure 5:**
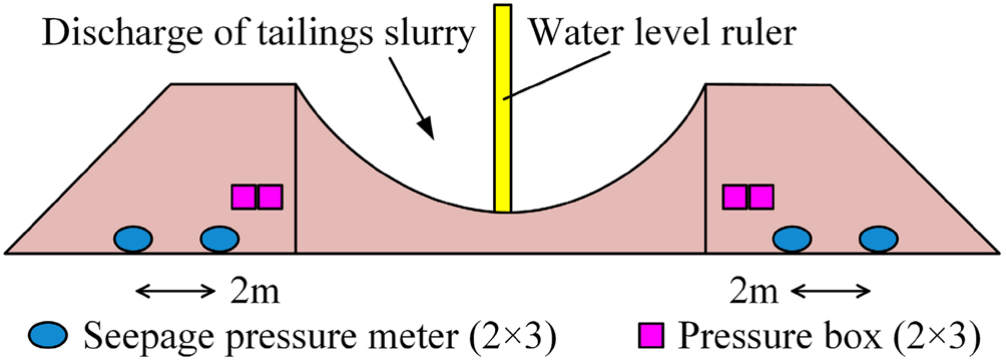
Deployment of field measurement instruments.

The main field test monitoring instruments were pore water pressure monitoring instruments (seepage pressure meters) and internal soil pressure monitoring instruments (soil pressure boxes). Two seepage pressure gauges were placed horizontally at 2 m intervals in the middle cross-section of each stacking dam, and one earth pressure box was placed horizontally and vertically in the middle cross-section of the stacking dam, as shown in Fig. 5.

#### Comparison between pore water pressure simulation value and monitoring value

**Figure 6:**
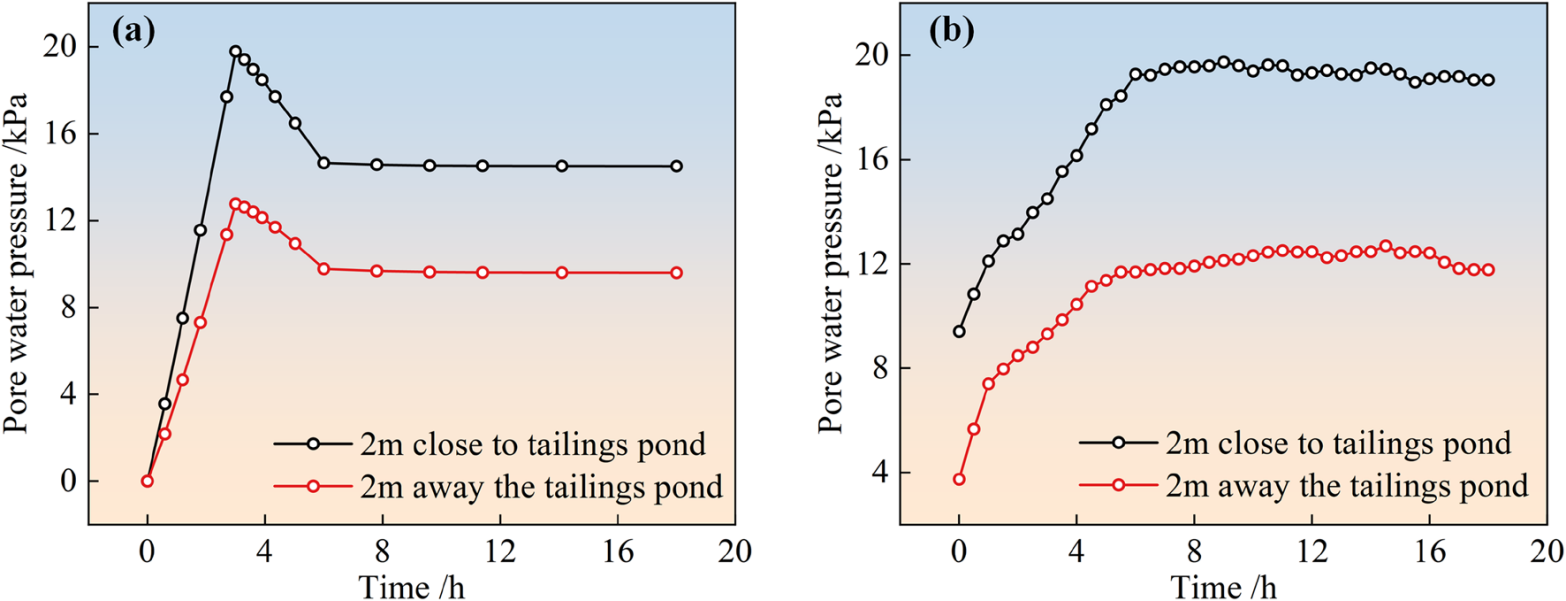
Simulation and monitoring of pore water pressure with time for conventional accumulation dams: (**a**) Simulation value; (**b**) Monitoring value.

**Figure 7:**
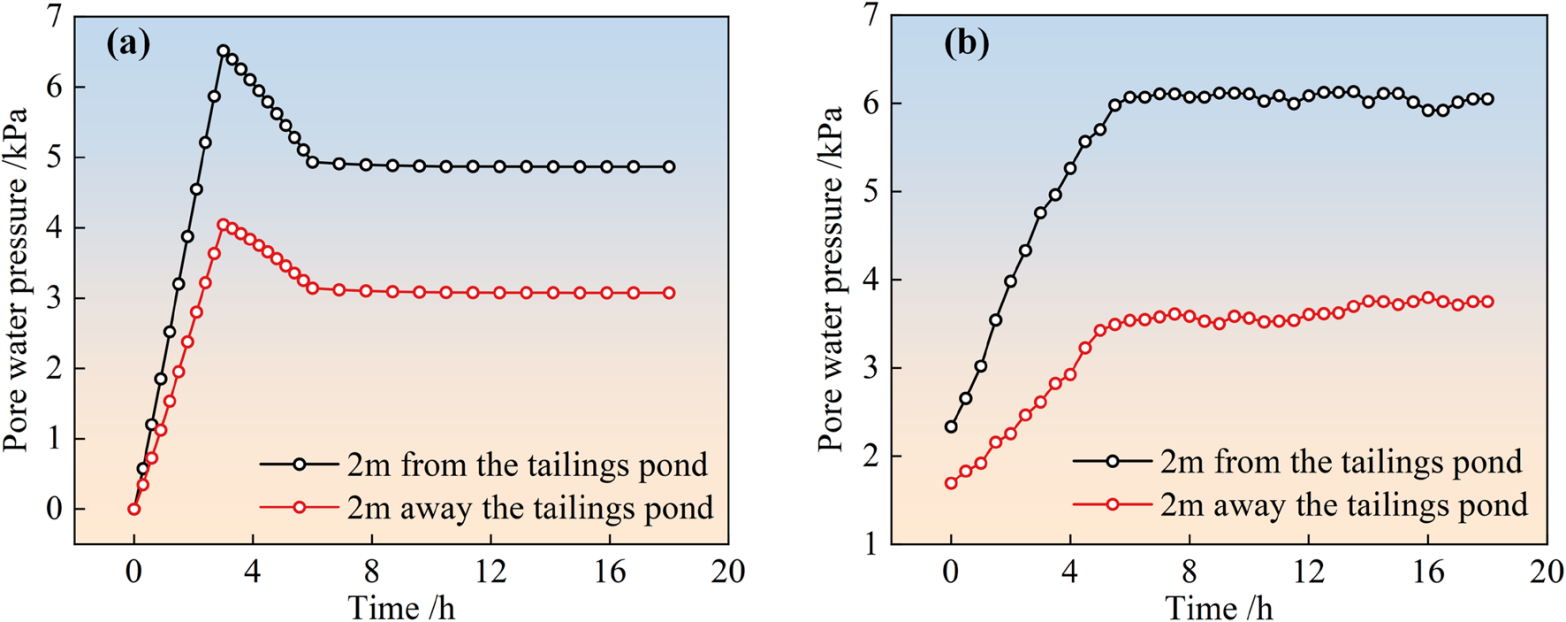
Simulation and monitoring of pore water pressure with time for geogrid reinforced accumulation dams: (**a**) Simulation value; (**b**) Monitoring value.

The simulated and field-monitored pore-water pressure variations over time for the two types of stockpile dams after slurry injection into the surrounding tailings dams are shown in Figs. 6 and 7, respectively. From the figure, it can be seen that the simulated and monitored values of the pore pressure inside the two types of stacked dams agreed with each other as a function of time. The variation laws were as follows: For the simulated pore pressure, the trend over time was basically the same as the trend of the water level in the reservoir of the surrounding stacked dam. That is, the simulated pore pressure increased linearly with time for 3 h before the start of the observation, gradually decreased linearly to a stable value after reaching a peak, and then tended to stabilize. For the monitored pore pressure, the pore pressure in the first 6 h of observation gradually increased with time and then tended to stabilize.

Further analysis of the variation in the dam pore pressure throughout the monitoring process was presented in Table 4. The simulated peak pore water pressure in the table was compared with the measured peak, and it can be seen that the error was 0.3–6.5%. That is, the simulated peak pore water pressure of the stacked dam closely matched the measured peak. This indicates that the calculation model and parameters determined by the inverse trial calculation are reasonable and could better match the actual situation. The final stable value of pore pressure of the conventional dam was the largest, and the stable value of pore pressure of the geogrid-reinforced dam was not much different, indicating that reinforcement had a significant effect on the “resistance” of pore pressure inside the tailings dam. Reinforcement of tailings dams could effectively reduce the water level inside the dam body. This was because the reinforcement treatment of the tailings dam could make the reinforced tailings complex form a drainage prism, promoting the discharge of water inside the dam body, and reducing the pore water pressure inside the dam body.

**Table 4:** Variation of pore water pressure inside the dam body.

Type of dam body	Conventional accumulation dams/kPa		Geogrid reinforced accumulation dams/kPa	
	Away the tailings pond	Close to tailings pond	Away the tailings pond	Close to tailings pond
Monitoring peak	12.52	19.74	4.04	6.52
Simulation peak	12.76	19.79	3.80	6.12
Error	2%	0.3%	6.3%	6.5%

#### Comparison of earth pressure simulation and monitoring values

**Figure 8:**
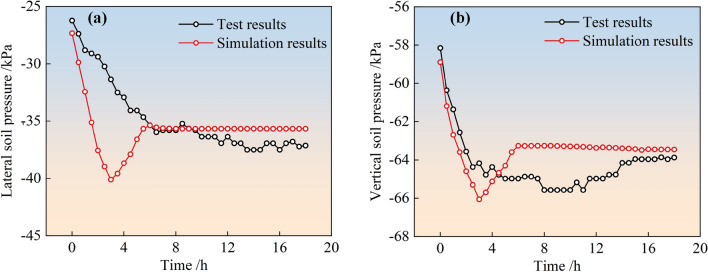
Simulation and monitoring of soil pressure with time for conventional accumulation dams: (**a**) Lateral soil pressure; (**b**) Vertical soil pressure.

**Figure 9:**
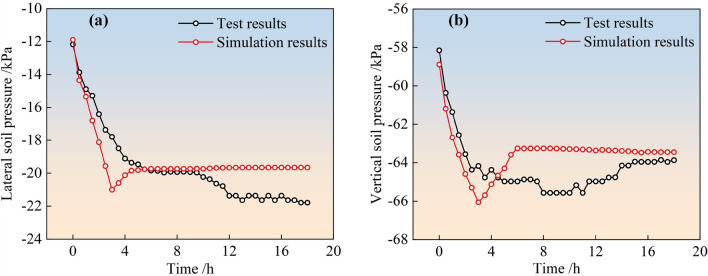
Simulation and monitoring of soil pressure with time for geogrid reinforced accumulation dams: (**a**) Lateral soil pressure; (**b**) Vertical soil pressure.

The simulated and field-monitored values of earth pressure (lateral and vertical earth pressure) over time for the two types of stockpile dams after slurry injection into the surrounding tailings dams are shown in Figs. 8 and 9, respectively. From the figure, it can be observed that the simulated and monitored values of the soil pressure inside the two types of stockpile dams are consistent with each other as a function of time, and the variation patterns are as follows. For the simulated soil pressure, its trend with time is basically consistent with the trend of the water level in the reservoir of the surrounding pile dam. That is, the lateral soil pressure and vertical soil pressure both increased linearly with time continuously in the first 3 h of observation, reached a peak, gradually decreased linearly to a stable value, and then tended to stabilize. For monitored soil pressure, both transverse and vertical soil pressure gradually increased with time and then stabilized, but transverse pressure changed more drastically compared to vertical soil pressure before stabilization.

Further analysis of the changes in the dam earth pressure throughout the monitoring process was presented in Table 5. According to the table, the simulated peak soil pressure inside the two types of dams basically matched the monitored peak with an error between 1.7 and 6.9%. This indicated that the calculation model and parameters determined by the inverse trial calculation were reasonable and can better matched the actual situation.

**Table 5:** Variation of soil pressure inside the dam.

Type of dam body	Conventional accumulation dams/kPa		Geogrid reinforced accumulation dams/kPa	
	Lateral soil pressure	Vertical soil pressure	Lateral soil pressure	Vertical soil pressure
Monitoring peak	37.5	64.97	21.78	45.95
Simulation peak	40.10	66.05	20.96	44.89
Error	6.9%	1.7%	3.9%	2.4%

The possible reasons for the errors in pore and earth pressure simulation and detection values are as follows: (1) There was a deviation between the position of the output point of the simulated time field and the position of the measuring point in the test; (2) The osmometer or earth pressure box in the test was displaced; (3) The field test was affected by the environment, weather, and other factors, leading to a deviation between the simulated and actual working conditions; (4) The mesh generation was not accurate in the process of establishing the model.

#### Determination of permeability parameters.

After the above analysis, the numerical value calculated by the calculation model determined by the trial calculation was consistent with the actual situation. Therefore, the determination of model parameters was reasonable, and the permeability coefficients of the dam body and geogrid were determined to be 2.0 × 10^−5^ and 1.8 × 10^−4^ cm s^−1^, respectively.

### Experimental optimization of geogrid interface characteristics

**Figure 10:**
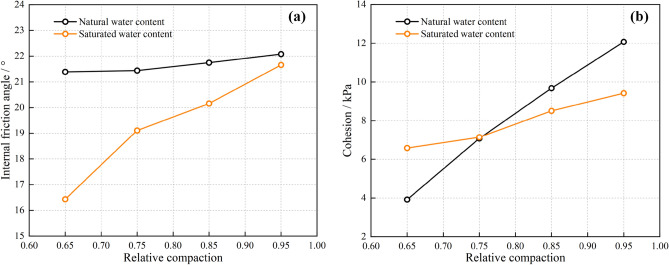
Interface characteristics of geogrid: (**a**) Internal friction angle; (**b**) Cohesion.

The main influencing factors in the construction process of a reinforced tailings dam were the water content and relative density. Therefore, the relative density and water content were used as the influencing factors to optimize the model further, and the YT1200 direct shear drawing test machine was used for the test. According to the compaction test and drying test, the relative density of tailings under natural conditions was 0.6, and the natural moisture content was 6.4%. Four working conditions with relative densities of 0.65, 0.75, 0.85, and 0.95 were selected, and the water content was natural and saturated water content (20.8%). The normal stress was determined to be 10 kPa, 20 kPa, 30 kPa, and 40 kPa through the preliminary examination to avoid the geogrid being pulled off during the test. The test results are shown in Fig. 10.

It was observed from the figure that when the relative density increases from 0.65 to 0.95, the curves of internal friction angle and cohesion show a monotonically increasing trend regardless of natural water content or saturated water content. With the increase of relative density, the shear strength under saturated water content was lower than that under natural water content. In summary, in the modeling of the geogrid reinforced tailings dam, the water content of tailings should have been determined by test to determine the shear strength parameters of the tailings dam. In the absence of test conditions, the interface shear strength parameters of saturated tailings below the saturation line should have been selected at a lower value, which was related to the stability and safety of the reinforced structure.

## Establishment of finite element model of tailings dam

Taking the main dam of the Fengshuigou tailings reservoir as the engineering background, the current elevation of the tailings reservoir reached close to 160 m, which approached the design elevation. Currently, the mining company intended to extend its service life and planned to expand its capacity to a design elevation of 175 m. At this time, the height of the accumulation dam was 100 m, the maximum dam height reached 120 m, the calculated total storage capacity was approximately 68,443 million m^3^, and the tailings pond was classified as the first grade. The initial dam of the main dam of the Fengshuigou tailings reservoir was a permeable rockfill dam. The dam crest width was 4 m, the upstream slope ratio was 1:1.85, the downstream slope ratio was 1:2, the dam bottom elevation was 55 m, the dam crest elevation was 75 m, and the dam height was 20 m. There was a 4 m wide horse road at an elevation of 66 m on the outer slope of the initial dam, and the dam foundation was located on the gravel layer. The accumulation dam was constructed upstream. The height of each sub-dam was 5 m. The ratio of the inner and outer slopes of the sub dam was 1:2. The top width of the sub-dam was 15 m. The average outer slope ratio of the accumulation dam was 1:5. There was a 50 m wide platform at an elevation of 120 m.

### Finite element model generalization

**Figure 11:**
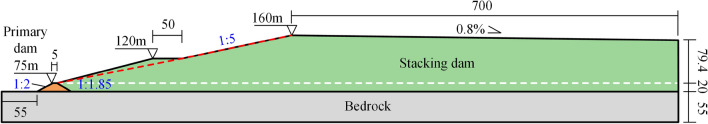
Calculation diagram of the current situation of the main dam of the tailings pond (Unit: m).

**Figure 12:**
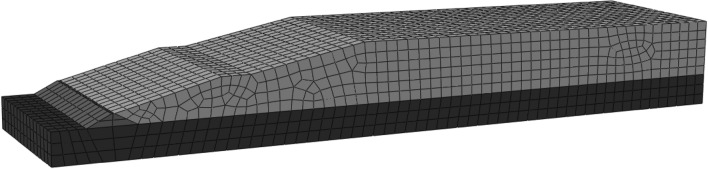
The current finite element model of the main dam of the tailings pond.

According to the tailings pond site survey information, the tailings dam was generalized, and the heightened dam body will be extended according to the generalized layering line of the current dam body. Therefore, considering the most dangerous situation during the simulation, drainage wells and other auxiliary facilities were ignored. The simplified and generalized calculation diagram is shown in Fig. 11, and the simulation diagram is shown in Fig. 12.

### Hypothetical conditions

In the design of the reinforcement scheme, it was assumed that^33^:

- The tailings dam was in normal operation, and there were no unexpected natural disasters such as heavy rainfall, mountain torrents, and earthquakes in the area.
- The geogrid and filler were closely meshed and embedded, there was no relative slip with the soil, and deformation coordination was maintained.
- The mechanical properties of the geogrid were intact; the geogrid was free from damage, fracture, and loose splicing during construction and laying.

### Model parameter selection

The tailings stockpile dam model was appropriately simplified in the finite element model, and the tailings stockpile dam was considered to have been formed by the accumulation of fine-grained tailings. The composition of the main dam profile and the physical and mechanical parameters of the geogrid are listed in Table 6.

**Table 6:** Physical and mechanical parameters of materials.

Materials	Unit weigh/(kN m^−3^)	Internal friction angle/°	Cohesion/kPa	Permeability coefficient/(cm s^−1^)	Elastic modulus/kPa	Poisson’s ratio
Primary dam	25	38.5	9.4	0.23	3.5 × 10^5^	0.35
Totally tailings	15.5	31.8	1	2.0 × 10^−5^	1.6 × 10^5^	0.25
Bedrock	27	42	21	1 × 10^−7^	6.0 × 10^7^	0.22
Geogrid	0.78	–	–	1.8 × 10^−4^	2.5 × 10^9^	0.29

### Boundary conditions and constitutive model

The three sides of the tailings accumulation dam were surrounded by mountains and belonged to a valley-type tailings reservoir. A finite element numerical model was established by considering the distribution of the tailings accumulation dam on the longitudinal section of the central axis. The boundary conditions were as follows: the displacement of the dam bottom was fixed in three directions, the normal displacement of other surfaces was constrained, the upper end was the free boundary, the 55 m elevation head was set at the bottom of the primary dam, and the upstream elevation head was set according to the length of the main dam dry beach.

The Mohr–Coulomb elastoplastic model was applied to the constitutive model of rock and soil; the elastic model was selected for the reinforcement, in which the section properties of the grid and the section properties of the rock and soil are entities. The soil steady-state analysis step was adopted. Because the constitutive model of the rock and soil was the Mohr–Coulomb model, the asymmetric unsymmetric algorithm was used to analyze and calculate.

### Calculation conditions

**Figure 13:**
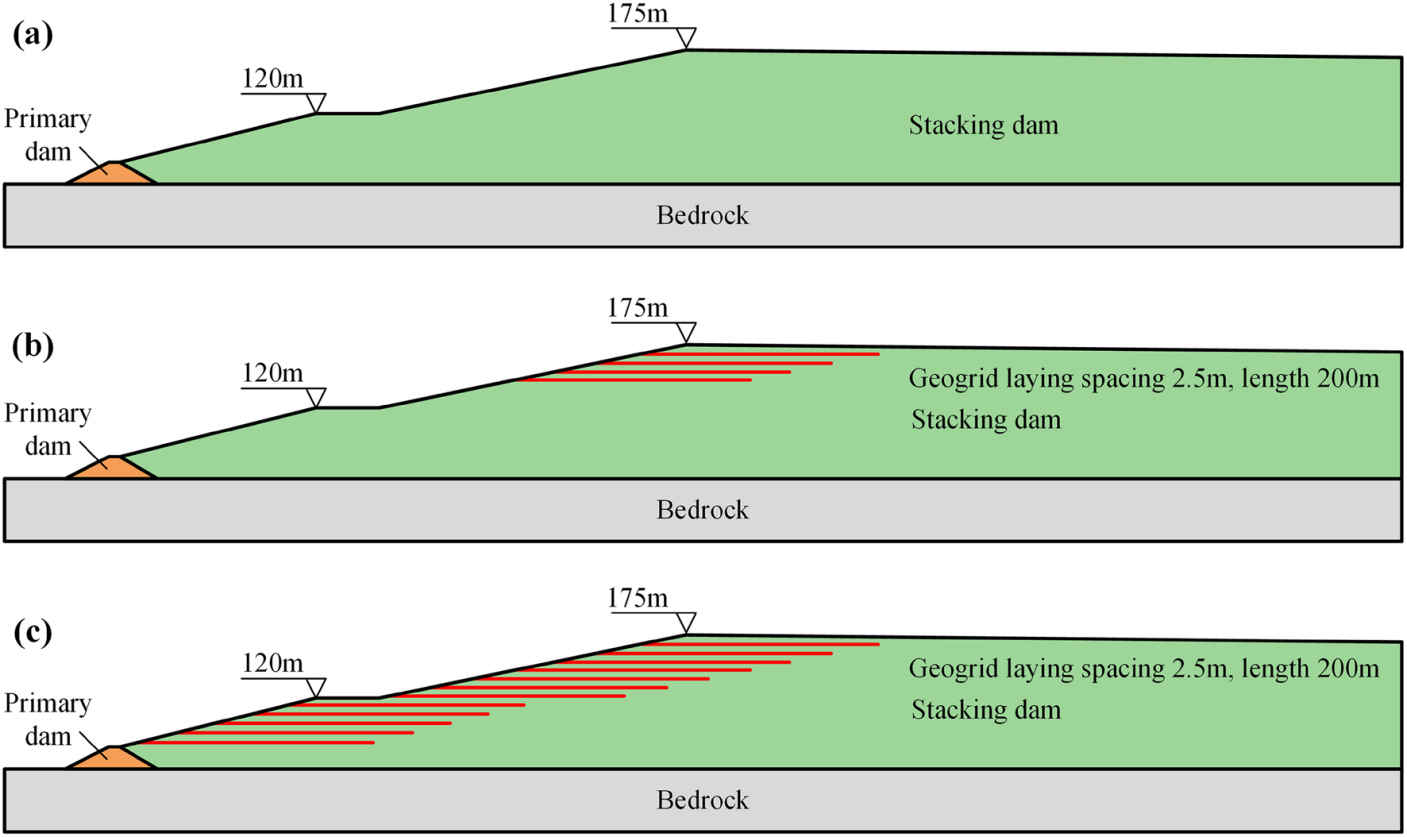
Three types of main dams when the final design elevation is 175 m: (**a**) Unreinforced; (**b**) Reinforcement of the 160–175 m elevation section; (**c**) Reinforcement of the 75–175 m elevation section.

For calculations, we referred to the “code for design of tailings facilities”^34^ (GB 50863-2013) and “code for seismic design of structures”^35^ (GB 50191-2012), carried out simulation calculations according to the following conditions, and compared and analyzed the changes of saturation line position and safety factor of the main dam under different working conditions:

- First, according to the current situation and future design plan of the tailings dam, and considering that the heightening affected the seepage field and stability of the tailings dam, the current elevation of 160 m and the final design elevation of 175 m were considered (See Figs. 11 and 13a);
- Second, to analyze the influence of reinforcement on the state of the tailings dam, considering the reinforcement of 160–175 m elevation section and 75–175 m elevation section, geogrid was selected as the reinforcement; it was only laid in the accumulation dam, the grating laying length was 200 m, and the spacing was 2.5 m (Fig. 13b,c).
- Third, considering the change in the water level in the tailings pond, the length of the dry beach was calculated by selecting the minimum length of the dry beach, flood operation, and normal operation (refer to Table 7 for the length of the dry beach at different elevations).

**Table 7:** Tailings pond dry beach length and safety superelevation requirements.

Tailings dam elevation	Specification limits/m		Flood operation/m		Normal operation/m	
	Minimum dry beach length	Minimum safety superelevation	Dry beach length	Safety superelevation	Dry beach length	Safety superelevation
Elevation 160 m	105	1.0	> 300	> 1.0	> 500	> 4.0
Elevation 175 m	120	1.0	> 300	> 1.0	> 500	> 4.0

Based on the above analysis, this simulation was carried out under the following four working conditions:

1. *Working condition I* current main dam elevation (160 m);
2. *Working condition II* final design elevation of the main dam without reinforcement (175 m);
3. *Working condition III* local reinforcement of the main dam at the final design elevation (160–175 m);
4. *Working condition IV* overall reinforcement of the main dam at the final design elevation (75–175 m).

## Results and analysis

### Saturation line analysis

**Figure 14:**
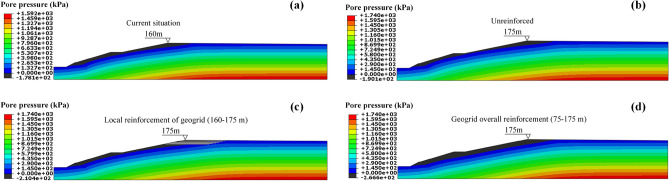
Cloud chart of pore pressure distribution at minimum dry beach length under different working conditions: (**a**) Working condition I; (**b**) Working condition II; (**c**) Working condition III; (**d**) Working condition IV.

As shown in Fig. 14, under the four minimum dry beach working conditions of 160 m, 175 m without reinforcement, 160–175 m with geogrid, and 75–175 m with geogrid, the distribution of the saturation line when the pore pressure of the tailings dam is 0. The seepage free surface of the tailings dam is curved, and negative pore water pressure appears in the upper part of the saturation line. This area is unsaturated, which is beneficial for the stability of the dam body. The pore pressure at the bottom of the saturation line was positive and saturated. The pore water pressure of the dam body was evenly distributed, and the pore pressure increased gradually from top to bottom. The outlet point of the saturation line of the dam body was near the dam body at the foot of the upstream slope of the primary dam. There is no intersection with the dam surface of the tailings accumulation dam, and groundwater does not overflow from the dam surface.

**Figure 15:**
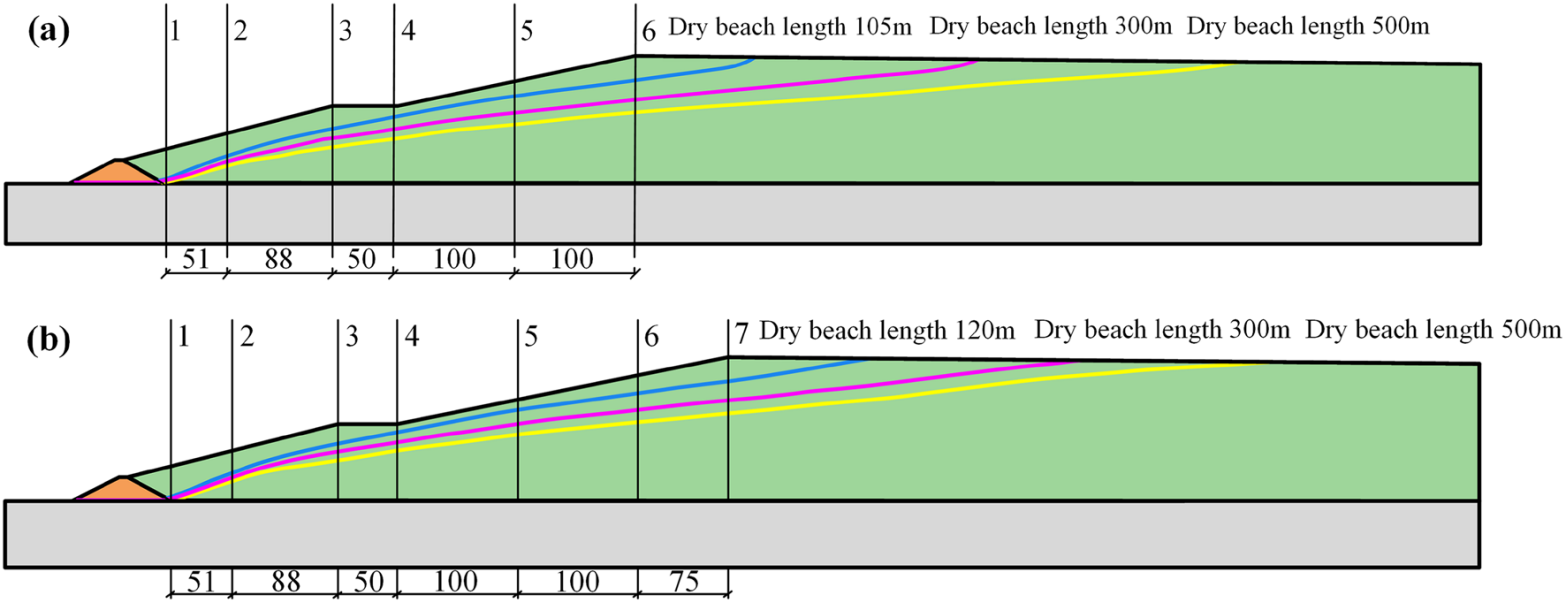
Saturation line trend and profile layout diagram with different dry beach lengths: (**a**) Working condition I; (**b**) Working conditions II, III and IV.

**Figure 16:**
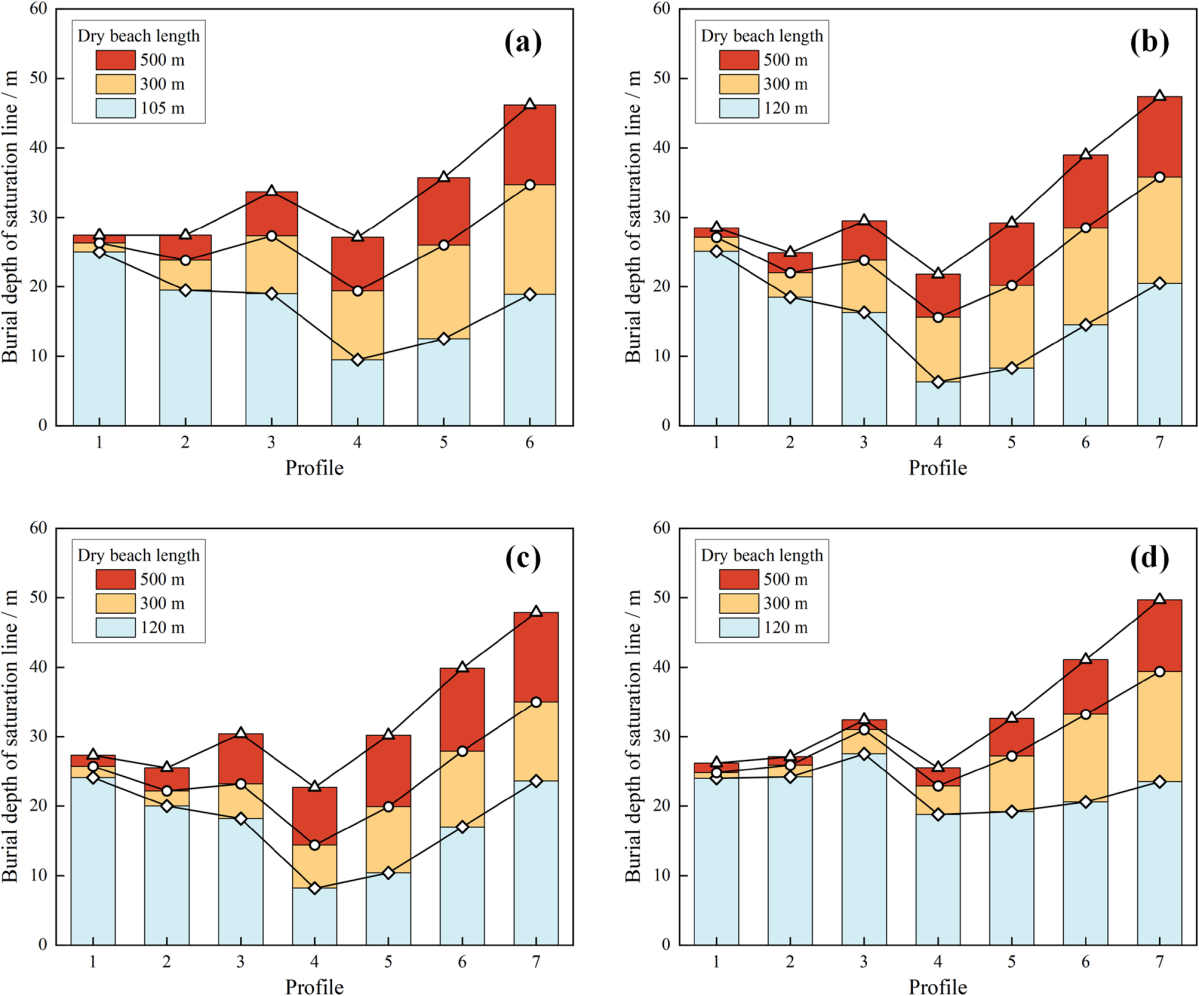
Burial depth of saturation line of each section of dam body under different working conditions: (**a**) Working condition I; (**b**) Working condition II; (**c**) Working condition III; (**d**) Working condition IV.

As shown in Fig. 15, six or seven profiles were selected from the slope of the accumulation dam for analysis. The buried depth of the saturation line from each profile is listed according to the pore pressure distribution, as shown in Fig. 16. Under the four working conditions, when the minimum dry beach was reached, the minimum buried depth of saturation line in each profile was 9.5 m, which was more than 6 m; therefore, it met the requirements of the “code for seismic design of structures” (GB50191-2012), which states that "the buried depth of saturation line on the downstream slope of grade I tailings dam should not have been less than 6 m.” As the length of the dry beach grows, the gradient of the saturation line slowed down, the path became longer and the position of saturation line in the upstream part of the dam body was significantly reduced; however, near the downstream primary dam, the location of the seepage point of the saturation line also decreased, although the change was small.

### Safety factor analysis

**Figure 17:**
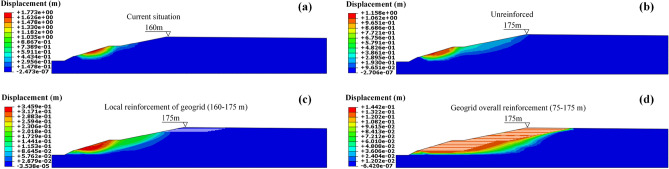
Incremental displacement nephogram for minimum dry beach length under different working conditions: (**a**) Working condition I; (**b**) Working condition II; (**c**) Working condition III; (**d**) Working condition IV.

Under the four minimum dry beach conditions (current 160 m, no reinforcement 175 m, local reinforcement of geogrid 160–175 m, and overall reinforcement of geogrid 75–175 m), the incremental displacement cloud diagram of the main dam was shown in Fig. 17. It can be observed from the incremental displacement cloud diagram that the sliding surface of the main dam was circular when it was unstable, and the sliding surface appeared at the accumulation dam under the tailings dam platform. This occurred because it was easy for the site to concentrate groundwater to the location caused by displacement deformation and ultimately caused damage. Further analysis showed that with the increase in dry beach length, the depth of the sliding surface gradually became shallow, and the area of the sliding body gradually decreased.

**Figure 18:**
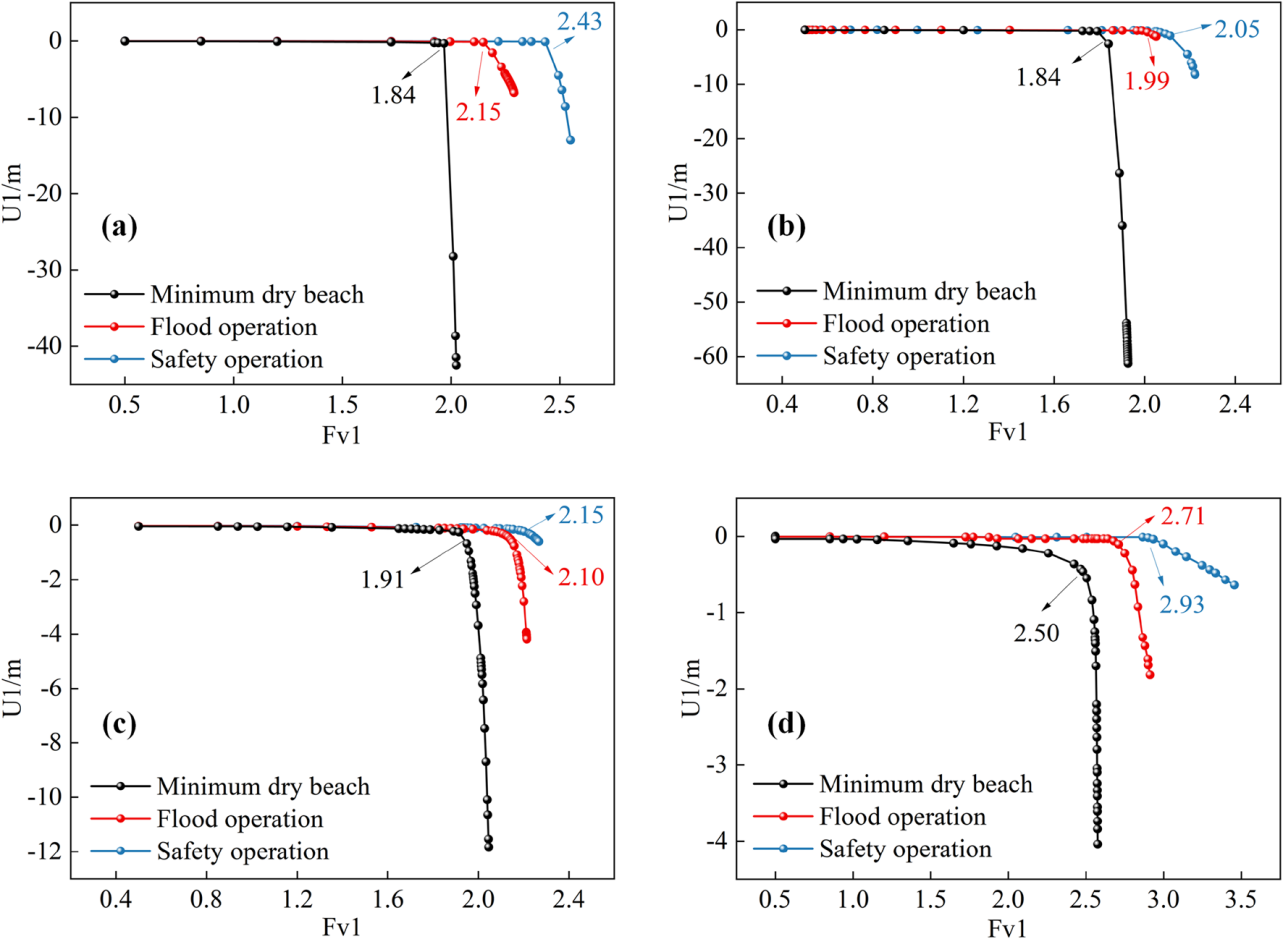
Variation of dam safety factor and displacement under different working conditions: (**a**) Working condition I; (**b**) Working condition II; (**c**) Working condition III; (**d**) Working condition IV.

In combination with the positioning of the sliding surface of the displacement nephogram, the top of the platform downhill of the accumulation dam was chosen as the characteristic control point, and the sudden change in the horizontal displacement of the characteristic control point was used as the criterion for the instability of the main dam. The relationship between the displacement of the characteristic points and the strength reduction coefficient under different dry beach lengths is shown in Fig. 18. When the current main dam was 160 m, the safety factors of the main dam under different dry beach lengths (minimum dry beach, flood operation, and normal operation) were 1.97, 2.15, and 2.43, respectively; when the elevation of the unreinforced main dam was 175 m, the safety factors of the dam body under different dry beach lengths (minimum dry beach, flood operation, and normal operation) were 1.84, 1.99, and 2.05, respectively. When the geogrid was only reinforced at an elevation of 160–175 m from the main dam, the safety factors of the dam slope under different dry beach lengths (minimum dry beach, flood operation, and normal operation) were 1.91, 2.10, and 2.15, respectively. When geogrid reinforcement was carried out at an elevation of 75–175 m from the main dam, the safety factors of the dam slope under different dry beach lengths (minimum dry beach, flood operation, and normal operation) were 2.50, 2.71 and 2.93, respectively.

### Calculation results verification

**Figure 19:**
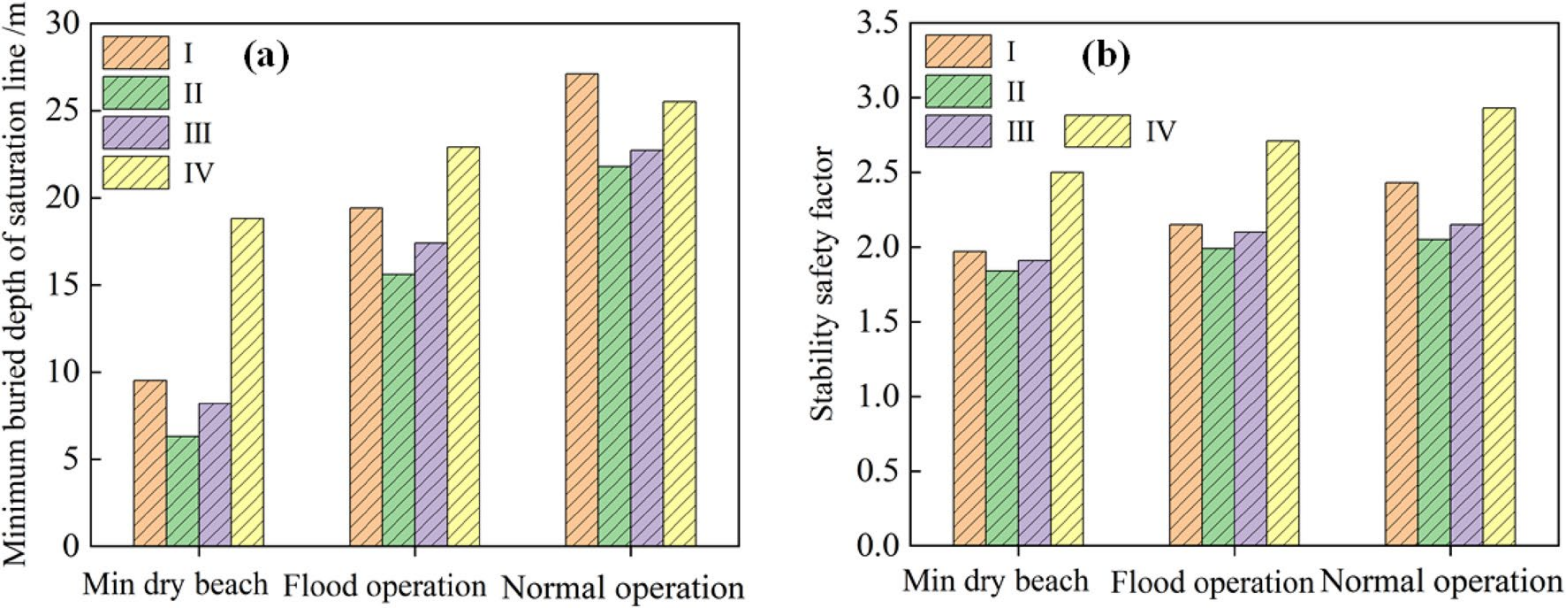
Comparison of calculation results of four working conditions under different dry beach lengths: (**a**) Minimum buried depth of saturation line; (**b**) Safety factor.

The simulation results under the three reinforcement conditions of 160 m, 175 m without reinforcement, 160–175 m with geogrid reinforcement, and 75–175 m with geogrid reinforcement are shown in Fig. 19. According to the “code for design of tailings facilities”^34^, the minimum safety factors for anti-sliding stability of a tailings dam during the minimum dry beach, flood operation, and regular operation were 1.30, 1.20, and 1.10, respectively. According to the safety factor results listed in Fig. 19, under two elevation conditions, the safety factor of the main dam ranged from 1.84 to 2.93 for different dry beach lengths. Consequently, the main dam was able to operate normally under different working conditions, and the safety reserve values were large.

When the main dam increased from 160 to 175 m, the minimum buried depth of saturation line decreased, that is, the saturation line of the main dam increased by approximately 2.8–5.3 m, which is unfavorable to seepage stability. Simultaneously, the safety factor was reduced by approximately 7–15.6%. Therefore, if the main dam was raised to 175 m, the length of the dry beach should be strictly controlled, that is, the water level in the reservoir should be strictly controlled, and the monitoring of the dam saturation line had to be strengthened to improve the comprehensive drainage capacity of the dam and reduce the water level in the reservoir and the dam saturation line position as much as possible.

Considering the actual situation, after geogrid reinforcement of the main dam from 160 to 175 m, the height of the saturation line decreased by 0–3 m, and the safety factor increased by 0–5.5% under three dry beach lengths. In the ideal state, geogrid reinforcement was carried out on the entire accumulation dam. Under the three dry beach lengths, the maximum decrease of the saturation line height reached 12.5 m, and the maximum increase of the safety factor reached 0.4 times. It could be seen that only the reinforcement at the elevation of 160–175 m of the accumulation dam could reduce the saturation line and improve the safety factor of the dam body, but the effect was not obvious. If the saturation line of the main dam was significantly reduced after the overall reinforcement of the accumulation dam, the harm of seepage could be greatly reduced, and the stability could be significantly improved. Therefore, this study could be referred to in practical engineering. After the construction of the initial dam of the tailings reservoir was completed, geogrid reinforcement was carried out on the accumulation dam.

### Comparative analysis of the results

In this paper, the stability of the tailings dam was studied using the finite element strength reduction method. To further verify the reliability of the simulation results, the limit equilibrium method was used, and the safety factor of the tailings dam under different working conditions was analyzed again by ABAQUS. The specific results are shown in Table 8. It can be seen from the table that the difference in the safety factor of the dam calculated by the two different methods is small. This is consistent with the research results of Cheng et al.^36^; that is, the calculation results of the limit equilibrium method were slightly smaller than the strength reduction method, and the errors of the two methods were within 5%. This shows that the strength reduction method used in this paper, combined with the results of ABAQUS simulation calculation, is reliable.

**Table 8:** Numerical simulation results under different working conditions.

Safety factor	Strength reduction method			Limit equilibrium method		
	Minimum dry beach	Flood operation	Normal operation	Minimum dry beach	Flood operation	Normal operation
Working condition II	1.84	1.99	2.05	1.75	1.90	1.97
Working condition III	1.91	2.10	2.15	1.82	2.01	2.07
Working condition IV	2.50	2.71	2.93	2.39	2.60	2.83

Compared with other research results on the safety factor of geogrid-reinforced slopes, Zhang et al.^37^ used FLAC3D software to compare the stability of geogrid-reinforced and unreinforced dam bodies. The results showed that the safety factor increased from 1.69 to 2.37, 40.70%. In the case of using the strength reduction method in this paper, the safety factor under the minimum dry beach condition increased from 1.84 to 2.50, from 1.99 to 2.71 under flood operation conditions, and from 2.05 to 2.93 under normal operation conditions, achieving 35.87%, 36.18% and 42.93% growth, respectively. In the case of the limit equilibrium method, the safety factor increased from 1.75 to 2.39 under the minimum dry beach condition, from 1.90 to 2.60 under the flood operation condition, and from 1.97 to 2.83 under the normal operation condition. The corresponding increases are 36.57%, 36.84%, and 43.65%, respectively. The results demonstrate that the calculation results of the two methods are consistent with the research results of Zhang et al., especially under normal operating conditions. This also indicates that the geogrid significantly improved the seepage stability of the tailings dam.

## Discussion

In this paper, Fengshuigou tailings reservoir was taken as an example. The finite element strength reduction method was employed to simulate and calculate using ABAQUS. The location and safety factor of the saturation line under different working conditions were compared and analyzed. The influence of dam height, geogrid reinforcement, and dry beach length on the stability of the tailings dam under seepage was discussed, providing reference for similar tailings dam projects.

### Analysis of tailings dam accident and practical significance of research

The problem of seepage stability led to the destruction of the tailings dam structure and eventually resulted in a dam break. Referring to the relevant data^38–40^, the probability of dam failure accidents due to flow sliding under various dam construction processes was shown in Table 9. It was observed from the table that, under the upstream dam construction process, the failure rate of tailings dam flow sliding was the highest, reaching 88%, significantly greater than other dam construction processes. As is known, a tailings dam break was a serious environmental disaster with potential long-term negative impacts on the surrounding environment and human society^1^. It caused significant losses to the local economy and led to casualties and disappearances of nearby residents. Therefore, the seepage stability of the tailings dam could not be ignored. The reinforcement of geogrid played an active role in improving the seepage stability of the tailings dam. It enhanced the shear strength of the dam, reduced the height of the saturation line, improved the safety factor, and thus reduced the risk of dam failure.

**Table 9:** Dam failure probability of flow sliding under different dam construction conditions.

Type	Quantity of dams	Frequency of malfunctions	Casualties	Average casualties	Failure rate (%)
Downstream	5031	3	0	0	6.00
Centerline	1583	2	0	0	4.00
Upstream	7926	44	1271	29	88.00
Other	3860	1	0	0	2.00

### Summary and prospect

From the modeling perspective, this paper only used the finite element strength reduction method for simulation and analysis, which had certain limitations. In the future, it could consider combining the spatial analysis ability of geographic information system (GIS)^41^ or use the soft computing technology of gene expression programming (GEP)^42,43^ for alternating modeling research. In summary, these factors would impact the stability of the tailings dam, which required more in-depth research in the future.

From the actual situation of the tailings dam, the tailings dam was generalized in the modeling process, which did not reflect the distribution of tailings dam materials in the actual state. The simulation effect had a specific error compared with the basic shape of the tailings dam, resulting in the limitation of the numerical simulation. In this study, when analyzing the influence of heightening and reinforcement on the seepage stability of tailings dams, only the effect of dry beach length, heightening, and geogrid reinforcement on the stability of tailings dams under the condition of fluid–solid coupling was studied. When considering the influence of geogrid reinforcement on the stability of tailings dams, only one grid laying method was assessed, and the effect of reinforcement spacing, reinforcement position, and reinforcement length was not considered.

From the point of view of the influence of consolidation on seepage, it was essential to consider the influence of consolidation on seepage in seepage analysis, especially when dealing with fine-grained soil and other materials with certain consolidation properties. Consolidation of tailings may occurred during loading or standing, mainly reflected in the following two aspects: First, the change of permeability coefficient. In the process of consolidation, the pore structure of the tailings changed, thus affecting the permeability of the tailings. The second was the change in pore water pressure. Consolidation resulted in the change of pore water pressure in tailings, which affected the driving force of seepage. In the seepage analysis of reinforced tailings dam, this paper used the finite element method to compare the simulated values of pore water pressure and earth pressure with the monitoring values, reasonably determined the permeability coefficient, and ensured the model’s accuracy. In the future, the corresponding consolidation model could be introduced to further improve the accuracy further.

## Conclusions

The seepage stability of the tailings dam could not be ignored. In this paper, ABAQUS software was used to simulate and analyze the influence of heightening and geogrid reinforcement on the stability of the tailings dam under different dry beach lengths. The following conclusions were drawn:

1. Geogrid played a positive role in reducing the saturation line of a tailings dam. In the case of dam heightening, the saturation line rose by 2.8–5.3 m. Compared with the unreinforced condition (dam heightening), the saturation line of the tailings dam under the local reinforcement condition was reduced by 0.9–2.8 m, and the saturation line of the tailings dam under the overall reinforcement condition was reduced by 3.2–12.5 m.
2. Geogrid also played a significant role in improving the stability of the tailings dam. In the case of heightening the dam, the safety factor was reduced by 7–15.6%. Compared with the unreinforced condition, the increase rate of the safety factor of the dam body under the local reinforcement condition was between 3.8 and 5.5%, and the increase rate of the safety factor under the overall reinforcement condition was between 35.9 and 42.9%.
3. The increase in dry beach length improved the stability of the tailings dam. The safety of the tailings dam under normal conditions was much higher than that under minimum dry beach conditions. Under the condition of the same dry beach length, the geogrid effectively improved the stability of the dam body compared with the unreinforced condition.
4. The fitting degree of the finite element model was good, and the error between the simulation and the experimental value was within 6.9%. This study provided a reference for the later heightening design of the main dam and the design of the new tailings reservoir, which had great reference significance for the design and operation of the reinforced tailings dam project.

## Data Availability

All data generated or analysed during this study are included in this published article.
